# Elevated *FMR1*-mRNA and lowered FMRP – A double-hit mechanism for psychiatric features in men with *FMR1* premutations

**DOI:** 10.1038/s41398-020-00863-w

**Published:** 2020-06-23

**Authors:** Andrea Schneider, Tri Indah Winarni, Ana María Cabal-Herrera, Susan Bacalman, Louise Gane, Paul Hagerman, Flora Tassone, Randi Hagerman

**Affiliations:** 1 Medical Investigation of Neurodevelopmental Disorders (MIND) Institute, University of California Davis Health, Sacramento, CA USA; 2 Department of Pediatrics, University of California Davis School of Medicine, Sacramento, CA USA; 3 Center for Biomedical Research, Faculty of Medicine Diponegoro University Semarang, Semarang, Central Java Indonesia; 4 Research Group on Perinatal Congenital Malformations and Dysmorphology (MACOS), Universidad del Valle, Cali, Colombia; 5 Department of Biochemistry and Molecular Medicine, University of California Davis, Sacramento, CA USA

**Keywords:** Bipolar disorder, Clinical genetics, Autism spectrum disorders, Schizophrenia

## Abstract

Fragile X syndrome (FXS) is caused by a full mutation of the *FMR1* gene (>200 CGG repeats and subsequent methylation), such that there is little or no *FMR1* protein (FMRP) produced, leading to intellectual disability (ID). Individuals with the premutation allele (55–200 CGG repeats, generally unmethylated) have elevated *FMR1* mRNA levels, a consequence of enhanced transcription, resulting in neuronal toxicity and a spectrum of premutation-associated disorders, including the neurodegenerative disorder fragile X-associated tremor/ataxia syndrome (FXTAS). Here we described 14 patients who had both lowered FMRP and elevated *FMR1* mRNA levels, representing dual mechanisms of clinical involvement, which may combine features of both FXS and FXTAS. In addition, the majority of these cases show psychiatric symptoms, including bipolar disorder, and/or psychotic features, which are rarely seen in those with just FXS.

## Introduction

The cause of fragile X syndrome (FXS) is a lack or deficiency of the *FMR1* protein (FMRP). This occurs when an individual has a full mutation CGG-repeat expansion (>200 CGG repeats and methylated) in the 5′ non-coding portion of the *FMR1* gene. There is a range of FMRP deficiency related to CGG-repeat/methylation status, with those having size and/or methylation mosaicism generally producing higher FMRP levels than those with the full mutation and fully methylated alleles^1^. Females with the full mutation generally have higher FMRP levels than males, due to the presence of a second X chromosome, with overall expression depending on their X activation ratio (the percentage of cells with the normal *FMR1* allele on the active X chromosome)^2^. The IQ in those with FXS correlates with the level of FMRP as does the presence of the physical features associated with FXS^2–4^.

The premutation (55–200 CGG repeats) is associated with elevation of *FMR1* mRNA (mRNA)^5,6^ and the development of neurological problems associated with aging, including the Fragile X-associated tremor ataxia syndrome (FXTAS)^7,8^ and fragile X-associated premature ovarian insufficiency (FXPOI)^9^. The pathogenesis in premutation disorders is thought to be a RNA toxicity or gain of function mechanism related to the elevated mRNA^10^. However, neurodevelopmental problems including hyperactivity, anxiety, social deficits, and autism spectrum disorders (ASD) have been reported in a subgroup of children and adults with the premutation, particularly those who present with full clinical symptoms^11–16^. Some of these individuals with the premutation and neurodevelopmental problems have a mild deficit of FMRP^16^. The level of FMRP gradually decreases as the CGG repeat number increases in the premutation range^17^, leading to some features of FXS in the high end of the premutation (over 110 CGG repeats)^2,18,19^. The gradual lowering of FMRP with the increasing size of the premutation has also been documented in the knock in mouse model of the premutation^17^.

There are various factors that influence FMRP levels among individuals with alterations in their *FRM1*. For instance, individuals can present with size mosaicism which is when cells carry both FM and PM alleles, and/or methylation mosaicism which is when some cells carry unmethylated FM alleles^20^. These mosaicisms account for the difference in FMRP expression and why some male individuals with the FM may have some level of protein expression. Although individual carriers of the PM have typically unmethylated alleles, some may present with a percentage of cells with a methylated PM allele, especially in those individuals with allele sizes in the upper PM^21^. The unmethylated alleles in those with methylation mosaicism are transcriptionally active and potentially translated into FMRP, but the extent of protein produced is also affected by the negative correlation between CGG repeat size and FMRP expression. Additionally, there is evidence that shows that PM alleles present somatic and germline instability so an individual with the PM may present with different CGG repeat size alleles. This is thought to be due to agents causing DNA damage and consequently promoting the CGG size expansion^22^. Levels of FMRP are therefore influenced by several factors including CGG repeat size, methylation status, presence of mosaicism, and mRNA levels.

In our clinical experience we have encountered several patients who have demonstrated both an elevated mRNA level (at least two times normal) and a lowered FMRP level (below 70% of normal). These individuals typically have an unmethylated full mutation, mosaicism, or a high end premutation. They therefore experience a dual mechanism of involvement including the toxicity of the elevated mRNA in addition to a deficit of FMRP. Although their phenotype is similar to those with FXS, there are also some additional features to the phenotype that are not typically seen with FXS alone without elevated mRNA. These additional phenotypic features can include early onset of neurological symptoms and /or psychotic ideation, and are described in detail in these cases.

## Method

We present a series of 14 male patients with different type of *FMR1* mutations seen at the University of California at Davis, MIND Institute. The visit included informed consent approved by the IRB at University of California Davis Medical Center. These cases were identified through the evaluation of fragile X families or direct referrals from affected family members. For each individual, the visit included a full medical history and examination, a neuropsychological assessment for intelligence, autism, and adaptive behavior. For the psychiatric diagnosis, the clients and/or their caregivers were assessed with a structured interview (Kiddie-SADS for children and adolescents, or the SCID, Structured Clinical Interview for DSM-IV Axis I Disorders for adults), some cases were interviewed with the ADIS (Anxiety Disorders Interview Schedule for DSM-IV: Lifetime version), or we obtained medical releases for the evaluation from the client’s outside psychiatrist or physician. The SCID-I and KSADS were administered by a psychiatrist, a clinical psychologist or licensed clinical social worker. The clinical ratings on the psychiatric interviews are reported as “threshold” – the patient meets full criteria of the disorder, or “subthreshold” – the patient only meets a subset of the symptoms, and not the full clinical criteria.

## Molecular analysis

### CGG repeat size

Genomic DNA was isolated from peripheral blood lymphocytes using standard methods (Qiagen, Valencia, California). Repeat size and methylation status were determined using both polymerase chain reaction and Southern blot analysis using an Alpha Innotech FluorChem 8800 Image Detection System (San Leandro, California) as previously described^23,24^.

### *FMR1* mRNA

All quantifications of *FMR1* mRNA were performed using a 7900 Sequence detector (PE Biosystems, Foster City, California) as previously described^5^.

### FMRP levels

Fragile X mental retardation protein was quantified in lymphocytes utilizing a sandwich enzyme linked immunosorbent assay (ELISA) for FMRP, as described previously^25^ as the ELISA approach provides a quantitative measure of FMRP level, whereas the immunocytochemistry method did not measure protein level, only the proportion of cells with detectable staining. The levels of FMRP in males with alterations in the FMR1 gene are reported as a percentage of the mean levels calculated for control males.

### Statistical analysis

FMRP and FMR1mRNA data were analyzed using univariate models. Since for some cases there was no FMRP data, a linear regression and missing value analysis (MVA) was calculated with SPSS 22.0 (Software Package for the Social Sciences). This method includes defining predictors of the variable (FMRP) with missing values using a correlation matrix. The best predictors are selected and used as independent variables in a linear regression model. The variable with missing data (FMRP) is used as the dependent variable. Cases with complete data for the predictor variables are used to generate the regression equation. This equation predicts missing values for incomplete cases. In an iterative process, values for the missing variable are inserted and then all cases are used to predict the dependent variable. MVA was performed with and without listwise deletion to compare the best fitting outcome.

## Results

Table 1 shows a summary of the cases including molecular data and psychopathology.

**Table 1:** Demographics, molecular results, and psychiatric features.

Case	Category	Age	CGG repeats	mRNA (SD)	FMRP	Full scale IQ	Dominant psychiatric feature
1	A	9	150	2.56 (0.27)		86 (WISC-IV)	Mood instability, ADHD
2	C	53	180–690	3.85 (0.30)		101 (WAIS-III)	Bipolar disorder NOS
3	B	36	205	2.59 (0.1)	6.04	51 (WAIS-III)	ADHD, schizophrenia, ID
4	B	28	226 (260–445)	3.85 (0.43)		117 (WAIS-III)	Social phobia, OCD, schizotypical symptoms
5	C	58	20–800	2.98 (0.06)		84 (WAIS-IV)	Bipolar I, OCD, panic disorder, generalized anxiety
6	B	25	570, 730, 1090 (240)	3.3 (0.04)	33.75	61 (WAIS-III)	ASD, Social Phobia, auditory hallucinations, ID
7	B	22	255 (270–535)	5.79 (0.13)	47.33	65 (WASI)	Psychosis in past, anxiety disorder, OCD, ID
8	B	22	215, 900 (220, 335)	2.30 (0.16)	13.42	45 (WISC-IV)	Social phobia, ASD symptoms, ID
9	D	23	160–190	5.02 (0.49)	70.00	86 (WISC-III)	ASD, psychosis
10	D	19	170	4.98 (0.32)	36.00	54 (WAIS-III)	ASD, paranoid thinking, anxiety disorder, ID
11	D	57	133	3.59 (0.28)			ASD, past ADHD
12	D	24	67	2.40 (0.23)	18.30	110 (WAIS-III)	ASD, bipolar II disorder, OCD
13	C	27	570, 750, 890 (104)	2.38 (0.05)		60 (SB5)	Anxiety disorder, ID, “socially awkward”
14	D	34	88	2.8 (0.18)		107 (WAIS-III)	Biploar I disorder, substance abuse, stress disorder, past ADHD

(A) Methylated Premutation, (B) Methylation Mosaicism, (C) Size/Methylation Mosaicism, (D) Premutation.

Under CGG repeats column the parentheses enclose the allele sizes that are unmethylated.

Normal *FMR1*mRNA range: 1.42 ± 0.26 (Tassone et al.^5^).

FMRP levels given in percentage of the mean levels calculated for control males.

data not available.

*ASD* autism spectrum disorder, *ADHD* attention deficit/hyperactivity disorder, *ID* intellectual disability, *OCD* obsessive compulsive disorder, *NOS* not otherwise specified, *SB5* Stanford Binet Intelligence Scales 5th edition, *WASI* Wechsler Abbreviated Scales of Intelligence, *WAIS* Wechsler Adult Intelligence Scale, *WISC* Wechsler Intelligence Scale for Children.

The mean age of the sample was 30.6 years (SD 14.55) with a mean IQ of 75.28 (SD 26.511). The average *FMR1* mRNA in the sample was ~3.86-fold above normal levels.

**Fig. 1:**
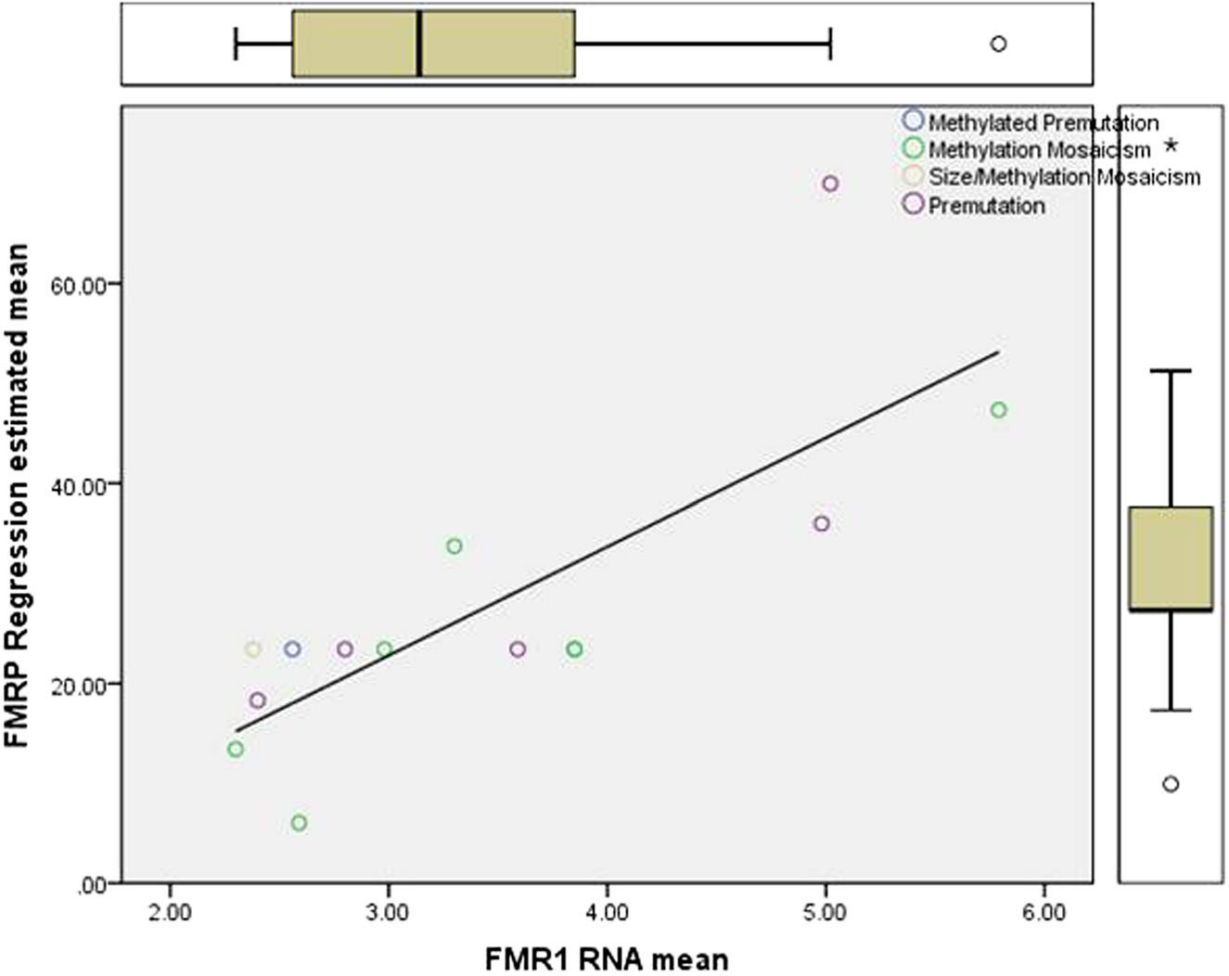
Regression model with a plotted linear fit line used for the Missing Values Analysis (MVA) for FMRP. The x-axis shows the *FMR1*mRNA mean, y-axis the FMRP Regression estimated mean, with standard deviation and mean plots to indicate the average value. The molecular status of an individual in the sample is plotted with a colored circle.

Because of the missing FMRP data, we calculated a linear regression and missing values analysis (MVA) with SPSS 22.0 (Software Package for the Social Sciences). Since half of the FMRP values were missing, the error terms were chosen randomly from a normal distribution. Figure 1 shows the regression graphics with a plotted linear fit line. Table 2 shows the univariate statistics for the FMRP and *FMR1*mRNA data. From this data, the MVA results and standard deviations are given in Table 3. The MVA regression mean for FMRP was 23.43, with an *FMR1* mRNA MVA of 3.45-fold above normal levels.

**Table 2:** Univariate statistics for FMRP and *FMR1*mRNA of the molecular data.

	*N*	Mean	Std. deviation	Missing	
				Count	Percent
FMRP	7	32.1196	22.00200	7	50.0
*FMR1* RNA	14	3.4564	1.12277	0	0.0

**Table 3:** Summary of estimated means and standard deviations (SD).

	FMRP	*FMR1* RNA	FMRP SD	*FMR1* RNA SD
Listwise deletion	32.1196	3.7686	22.00200	1.45820
Regression	23.4387	3.4564	20.71539	1.12277

### Case 1

Case 1 was a 9-year-old Caucasian boy with a premutation allele of 150 that was 10% methylated. Early developmental milestones were slightly delayed and he had a history of typical FXS symptoms such as hand flapping, tactile defensiveness, hyperactivity, perseveration, recurrent otitis media, severe anxiety, and tantrums. He had severe problems with mood instability that required risperidone since age 2 and then transition to aripiprazole at age 5. Sertraline had been helpful for his severe anxiety but stimulants had not been helpful for his severe ADHD symptoms. On the WISC-IV his Verbal Comprehension was 114, but his perceptual Reasoning was 90, Working Memory was 68, and Processing Speed was 70. On physical examination he had a slightly cupped right ear, doubled jointed thumbs, flat feet, and no tremor was observed. An ADOS Module 2 assessment the age of 5 did not show any signs of an autism spectrum disorder (ASD).

The psychiatric interview with the K-SADS was performed with the mother and a behavioral observation of the patient. The assessment showed a significant mood regulation problem with rigidity regarding going to bed-routines, frequent awakenings, bed-wetting, and food aversions with intensive gag reflexes. He still had severe tantrum, showed signs of hyperactivity and fleeting eye-contact. On the Behavior Assessment for Children, 2nd edition^26^ he scored in the 99th percentile on the depression subscale and the externalizing problems subscale; in the 98th percentile on the hyperactivity and aggression subscale, and on the atypicality subscale including problems such as “acting strangely”, and inappropriate affect.

### Case 2

Case 2 was a 53-year-old Caucasian male who had unmethylated alleles spanning from 180 to 690 CGG repeats. He had ADHD symptoms and significant impulsivity in childhood and after graduation from high school he began work as a carpenter. He developed severe hypertension at age 30 requiring antihypertensive medication. He developed an intermittent hand tremor that was noticeable when using tools or a pencil at age 49. He subsequently developed a tremor in his leg that feels as if a cell phone was vibrating in his pocket when there was none there. He experiences light-headedness and sometimes dizzy spells. On several occasions bright lights had flashed across his visual field although he did not have a history of migraines.

On his medical examination he had absent reflexes in all four extremities, a positive snout reflex, but no tremor nor ataxia were observed. The SCID revealed a diagnosis of Bipolar Disorder not otherwise specified (NOS) with the most current episode depressed, a history of heavy substance abuse (methamphetamine, alcohol, and stimulants), specific phobia, and a self-reported previous diagnosis ADHD with impulsivity. His MRI did not demonstrate the middle cerebellar peduncle sign which would be typical of FXTAS criteria.

### Case 3

Case 3 was a 36-year-old Caucasian male with a full mutation with methylation mosaicism. He was adopted at birth, and his birth mother had a history of drugs and alcohol. He reached his developmental milestones delayed (walking at 15 months, speaking single words at 18 months, phrase speech at age 5½ years). Upon medical examination, he demonstrated a long, somewhat narrow face. His palate was high-arched. His ears pinnae were not prominent but they were long measuring 7 cm in length. He was diagnosed with atypical autism at 5-years of age and had a history of ADHD problems. At the time of puberty he became more aggressive and his anxiety increased. According to the caregiver, he had two psychotic breaks at age 29 and 31 and was subsequently diagnosed with schizophrenia. He had been treated with aripiprazole and clonidine.

### Case 4

Case 4 was a 28-year-old Caucasian full mutation male with an almost complete lack of methylation. His developmental milestones were only slightly delayed. He is a high-functioning FXS male with great insight into emotions but he showed some Asperger Syndrome (DSM-IV) symptoms. On the Anxiety Disorders Interview Schedule^27^ interview, he met criteria for social phobia, and obsessive-compulsive disorder with overlapping psychotic-schizotypical (personality disorder) symptoms. His full scale IQ of 117 fell within the high average range.

In the SCID he reported frequent self-talk. He met clinical criteria for Social Phobia (with peers, strangers, in school). In the past he fulfilled criteria for depressive disorder (sub-threshold, only 2 main criteria out of 5 met). There was no evidence of mania current or past, but the he reported racing thoughts. Some symptoms point to prodromal features (peculiar behavior, impaired personal hygiene, inappropriate affect, and over-elaborate speech). In the past he met criteria for separation anxiety disorder (nightmares, physical symptoms, and excessive distress in anticipation of separations and separation, school reluctance, and fears of being alone at home) and Generalized Anxiety with strong self-consciousness.

### Case 5

Case 5 was a 58-year-old Caucasian male with an unmethylated full mutation. He had a long face with mildly prominent ears. He suffered from degenerative disc disease and chronic muscle pain and using an electric wheelchair. His full scale IQ of 84 fell within a low average range. On the SCID-I^28^ he met criteria for Bipolar I Disorder with a current episode of major depression. He met sub-threshold criteria for psychotic symptoms with hearing voices, and he did have some religious perseverations and grandiose thinking. He met criteria for panic disorder and generalized anxiety disorder. He had significant obsessive compulsive disorder (OCD) with sexual obsessions and frequent lock checking. On neurological examination he had an intention tremor, neuropathy symptoms, ataxia, while on physical examination he had macroorchidism (70 ml volume bilaterally) and a long face, which are features of the full mutation FXS. His MRI did not have the middle cerebellar peduncle sign but clinically, he appeared to have both FXS and FXTAS.

### Case 6

Case 6 was a 25-year-old Caucasian male with the full mutation. His developmental milestones were delayed with sitting at 7 months, crawling at 8 months, walking at 1 year, but at 2 years he was only saying single words and he did not speak in sentences until about 4 years of age. His behavior included hand flapping, poor eye contact, tactile defensiveness, hyperactivity, perseveration, tantrum behavior about three to four times a week, mainly with verbal outbursts, yelling and irritability at least once a day. His full scale IQ of 61 fell in a mildly delayed range. On the ADOS Module 4 he met ASD criteria. On the ADIS, he met criteria for social phobia, specific phobia, and generalized anxiety. He also showed some psychotic features with hearing voices, and laughs to himself frequently.

### Case 7

Case 7 was a 22-year-old Caucasian male with a full mutation with methylation mosaicism. At age 12 he showed intermittent psychotic symptoms with hearing voices in his head. At the last visit to our clinic at age 22, he had anger outbursts, attentional problems, and impulsivity and he also had a tendency to be a loner. He had some facilitated and repetitive speech, he might say “I hate you” or “I want to kill you,” which can be an automatic speech pattern typical of individuals with FXS. He showed several symptoms of an anxiety disorder and significant OCD with sexual obsessions. He had been treated with atypical antipsychotics including aripiprazole currently and risperidone in the past.

### Case 8

Case 8 was a 22-year-old Caucasian male with a low-level full mutation with methylation mosaicism that was 43% methylated. His developmental milestones were delayed with walking at ~19 months, saying words at 2½ years, but he did not speak in phrases until he was ~3½ years of age. He showed a lot of self-talk, perseverative speech, has difficulty in organizing his thinking, and can be quite tangential. His full scale IQ of 45 fell in a moderately delayed range. On the ADOS, he did not meet full clinical criteria for an ASD, but fell in the PDD-NOS category. On the ADIS he met clinical criteria for Social Phobia.

### Case 9

Case 9 was a Caucasian male with premutation of 160–193 CGG repeats. He met his early developmental milestones, but lost the ability to talk at 15 months which he later regained with speech therapy. At 2 years of age he suffered from multiple ear infections, a floppy body, hyperactivity, and hand biting. At 8 years, 6 months he displayed hand flapping, poor eye contact, tactile defensiveness, hyperactivity, anxiety, perseveration, aggression, and had daily outbursts. The physical examination showed prominent ears with slight forward cupping, slight pectus excavatum, and mild pronation. At age 12, while on Seroquel 25 mg, he became depressed and had at least 1 month of recurrent suicidal ideation where his mother had to lock up all the knives in the house. He was seen again at 13 years, 5 months of age. He was described as talkative and enthusiastic, but redirection was often required. On the ADOS, he scored an 11 and met overall criteria for an ASD. The WISC-III full scale IQ of 86 fell in a low average range. The caregiver reports severe dysphoric mood and irritability with rapid mood fluctuation. His speech was slightly pressured with normal volume and monotonous prosody. He had poor eye contact towards the parents and the interviewer. His thought process was coherent with evidence of magical thinking, including believing he could influence his mother’s actions through “ESP” (extrasensory perception) and could predict the Pokemon show. He said he had at least 20 different brains with different personalities that were responsible for his actions. At age 13 his psychiatrist diagnosed him with psychosis because he was hearing voices and hallucinating and his Seroquel was increased.

### Case 10

Case 10 was a 19-year-old Caucasian male with a premutation of 170 CGG repeats. His behavior included hand flapping at times when he was excited and jumping up and down. When he was 6 years old, he showed excessive laughing, significant ADHD, and aggression. He had some paranoid thinking about people making fun of him or staring at him, which continued to impair his social behavior. He was given the Kiddie-SADS and he met criteria for ADHD and generalized anxiety disorder, as well as autism. He showed some OCD symptoms with a sexual fetish. He also met criteria for autism on the ADOS Module 4 on the current assessment. His full scale IQ of 54 fell in a mildly delayed range.

### Case 11

Case 11 was a 57-year-old Caucasian male with a premutation of 133 CGG repeats. He had a history of ADHD and being socially awkward in childhood which led to a diagnosis of Asperger’s syndrome. At age 46 he began to experience cognitive decline leading to job loss, then an intention tremor began at age 47 and ataxia at age 50. He also had high blood pressure, moodiness, irritability and seizures treated with Keppra. On physical examination he had a somewhat long face, high-arched palate and prominent ear pinna with ear cupping bilaterally. On finger-to-nose touching he had a significant intention tremor, in addition to a postural tremor and ataxia with an inability to tandem walk. His MRI was consistent with FXTAS.

### Case 12

Case 12 was a 24-year-old Caucasian male with premutation of 67 CGG repeats. His developmental milestones were partially delayed with sitting at 13½ months, crawling at 14 months, walking at 16 months, saying words at 1 year, and phrases at ~30 months. He had a history of ASD in addition to psychiatric diagnoses including bipolar disorder, Tourette syndrome or chronic tic disorder, and OCD. He had also had four psychiatric hospitalizations in 2004 and twice in 2006. His full scale IQ was of 110. At age 24 he developed significant catatonia and was treated with several sessions of electroconvulsive therapy (ECT) with a good response. He had not tolerated antipsychotic medication well and he was eventually treated with lithium and clonazepam.

### Case 13

Case 13 was a 27-year-old Caucasian male with FXS mosaicism with 40% of his cells carrying the premutation of 104 CGG repeats, and the rest with a full mutation with partial methylation. His IQ at age 25 on the Stanford Binet showed a verbal IQ of 63, nonverbal of 60, and full scale IQ of 60. In his childhood he was anxious and socially awkward and he did well with atypical antipsychotics including aripiprazole and risperidone in addition to sertraline and topiramate. At age 24 he developed an oral motor dystonia with spasms of his tongue which made his speech unintelligible. He also developed a neck twisting movement. His risperidone was tapered without improvement of his symptoms and he was treated with a variety of medications including carbidopa/levodopa, and lamotrigine without effect, but he later improved with clonazepam and guanfacine.

### Case 14

Case 14 was a 34-year-old male with a premutation of 88 CGG repeats. He had a history of ADHD and a traumatic brain injury from jumping off a bed at 5 years old. He continued to have problems in school related to impulsivity, mood lability, ADHD and learning problems. In adolescence and adulthood he developed substance abuse problems including alcohol and cocaine. He spent time in rehabilitation before being evaluated at age 34. On the SCID, he met criteria for Bipolar Disorder I with the most recent episode manic, a history of Substance Abuse, and acute stress disorder. His Verbal IQ was 112, Performance IQ was 98, and full scale IQ was 107. He suffered from intermittent intention tremor that began in his late 20 s and long-term history of balance problems. He showed some autistic features. His MRI showed some white matter disease in the insula but no middle cerebellar peduncle sign. Because of his tremor and ataxia he had a possible diagnosis of FXTAS. After evaluation he subsequently began using alcohol again in addition to cocaine and methamphetamine. He developed paranoid ideation toward his mother and his father and threatened to kill them. He was subsequently shot and killed when he attacked someone without provocation.

## Discussion

We present 14 cases of individuals with *FMR1* mutations, including those with the premutation or mosaicism with a wide spectrum of psychiatric features. These cases are unusual because many cases include both features of FXS even though they are premutation carriers, or vice versa – patients with features of premutation involvement, even though they have a full mutation (some partially unmethylated or with methylation mosaicism). These cases have features of both FXS and premutation involvement that is straddling the border between these two different disorders with different pathophysiology. Lowered FMRP causes FXS, but in the upper range of the premutation, or even mid-level premutation carriers can have lowered FMRP due to less efficiency of protein translation^17,18^. The knowledge about medical and psychiatric problems associated with the premutation have increased significantly over the last decade to not only include FXTAS, but also include neuropathy, migraines, hypertension, sleep apnea, restless legs syndrome, anxiety, and depression^29–32^.

The cases presented here, however, have phenotypic features that go beyond what is usually seen FXS or premutation carriers alone. There is a high frequency of psychotic features that is unusual for FXS or premutation involvement. Some cases here also have the onset of neurological problems that begin much earlier than what is usually seen in premutation carriers who develop FXTAS at an average age of 62^33^. Instead the premutation carriers often have developmental problems that begin in childhood including ADHD and/or ASD. In addition, a decreased FMRP level underlies amygdala dysfunction that is associated with emotional processing^34–37^. This is most likely related to lowering of FMRP levels which occurs especially at the upper end of the premutation, although the developmental effects of RNA toxicity may also lead to developmental problems in some cases^14^. However, other environmental factors or epigenetic effects could influence the individuals presented in this case series, leading to different phenotypes and clinical features albeit similar genetic profiles on FMRP and *FMR1* mRNA levels.

Unifying criteria for all presented cases are affective disorder symptoms, mostly mood disorders, anxiety, and psychotic features. Given the high prevalence rate of the *FMR1* mutations, a clinician may have encountered patients who are carriers, already. Even though genetic/molecular testing may not be readily available, this case series aims to highlight the need for additional diagnostic considerations.

These phenotypic variations are important for the clinician to be aware of and to consider a double hit if the patient with a fragile X mutation presents with psychosis or early onset of neurological problems. FXTAS has been previously reported in individuals with FXS but in the previous cases they had a lack of methylation^38^ combined with alcohol abuse, and in another case there was mosaicism^39^ and both of these cases had a double hit. Substance abuse is not uncommon in carriers^40,41^ as was seen in our case 14. The lowering of FMRP associated with up-regulation of mGluR5 can also drive substance abuse^42,43^ so we believe a double hit is more likely to lead to such problems.

FMRP levels and mRNA levels are difficult to obtain on a clinical basis and there are a few cases in Table 1 that do not have an FMRP level. However, one can assume that any patient with a full mutation will have lowered FMRP, even with a lack of methylation. And any case with a CGG repeat above 120 will also have some degree of FMRP deficit^17,21^.

The neurological problems that may occur in those with FXS including the cases that have been described as FXTAS are still being investigated. Pretto et al.^44^ reported FXTAS inclusions in a mosaic male who died with a neurological condition that fit FXTAS but also had elements of Parkinson’s Disease (PD). PD has been reported in ~20% of aging males with FXS^45^, but the double hit may predispose a subgroup of patients to have earlier symptoms, particularly if they had developmental delay or ASD in childhood as in case 12. Further neuropathological studies are needed to better understand whether the neuropathology is the same for FXTAS like symptoms in those with FXS. Also the diagnostic criteria for FXTAS should be expanded to include those with >200 repeats particularly if there is a lack of methylation.

The association of low FMRP with psychotic features is becoming more intriguing since FMRP deficits correlate with earlier age of onset and lower IQ in those with schizophrenia without a *FMR1* mutation^46^. Further studies are definitely warranted regarding the association of psychosis and FMRP deficits especially in those with a double hit as reported here.
